# Characterising seasonal influenza epidemiology using primary care surveillance data

**DOI:** 10.1371/journal.pcbi.1006377

**Published:** 2018-08-16

**Authors:** Robert C. Cope, Joshua V. Ross, Monique Chilver, Nigel P. Stocks, Lewis Mitchell

**Affiliations:** 1 School of Mathematical Sciences, The University of Adelaide, Adelaide, South Australia, Australia; 2 Discipline of General Practice, The University of Adelaide, Adelaide, South Australia, Australia; 3 Stream Lead, Data to Decisions CRC, Adelaide, South Australia, Australia; University of California, Los Angeles, UNITED STATES

## Abstract

Understanding the epidemiology of seasonal influenza is critical for healthcare resource allocation and early detection of anomalous seasons. It can be challenging to obtain high-quality data of influenza cases specifically, as clinical presentations with influenza-like symptoms may instead be cases of one of a number of alternate respiratory viruses. We use a new dataset of confirmed influenza virological data from 2011-2016, along with high-quality denominators informing a hierarchical observation process, to model seasonal influenza dynamics in New South Wales, Australia. We use approximate Bayesian computation to estimate parameters in a climate-driven stochastic epidemic model, including the basic reproduction number *R*_0_, the proportion of the population susceptible to the circulating strain at the beginning of the season, and the probability an infected individual seeks treatment. We conclude that *R*_0_ and initial population susceptibility were strongly related, emphasising the challenges of identifying these parameters. Relatively high *R*_0_ values alongside low initial population susceptibility were among the results most consistent with these data. Our results reinforce the importance of distinguishing between *R*_0_ and the effective reproduction number (*R*_*e*_) in modelling studies.

## Author summary

When patients present to their doctor with influenza-like symptoms, they may have influenza, or some other respiratory virus. The only way to discriminate between these viruses is with an expensive test, which is not performed in many cases. Additionally, results other than influenza may not be reported. This means that it can be difficult to determine how much influenza is circulating in the population each season. We used a unique dataset of confirmed influenza with denominators to fit models for seasonal influenza in New South Wales, Australia. Knowing the denominators allowed us to estimate population level trends. We found that the relationship between influenza transmission rates and immunity due to previous infections was critical, with relatively high transmission corresponding to substantial preexisting immunity likely. This existing immunity is critical to understanding and effectively modeling influenza dynamics.

## Introduction

Influenza is a highly contagious, rapidly evolving respiratory virus that circulates globally on a seasonal basis [1, 2]. It can cause death in at-risk groups, and places a high burden on health systems (e.g., emergency room (ER) and general practitioner (GP) services). In some cases, antigenic shift creates circumstances where influenza can be particularly infectious or dangerous, resulting in influenza pandemics such as those that occurred in 1918 and 2009. However, understanding normal seasonal circulation is critical for effective healthcare resource allocation, forecasting future seasonal dynamics, and early detection of anomalous seasons.

Influenza has been widely studied, through both experimental studies on animal models such as ferrets (e.g. [3, 4]), and modelling studies based on data from human populations [5, 6]. There remains substantial uncertainty surrounding the way influenza spreads within populations. Model-based analyses depend on the underlying assumptions and available data, and knowledge gaps exist that may be difficult or impossible to test using animal models (e.g., for seasonal influenza, long-term patterns of immunity through and across many seasons). Parameter estimation can also be challenging. For example, a key epidemic parameter is the basic reproduction number, *R*_0_, which is defined using a completely susceptible population. However, in practice few populations are likely to be completely susceptible, and so estimates are instead of *R*_*e*_, the effective reproduction number [7, 8]. To determine *R*_0_ itself, it is necessary to also have an estimate of the proportion of individuals that are susceptible at the beginning of an outbreak. Unfortunately, estimating the initial susceptible proportion is close to impossible in practice, given the complexity of immunity and interactions between strains. Immunity to a strain may vary substantially between individuals with similar infection or vaccination histories [9], and the life course of antibodies and interactions between antibodies to different strains is complex [10]. The rapid evolution and multi-strain nature of influenza means that individuals may have variable immune responses to different strains, particularly in the presence of complex phenomena such as “antigenic seniority” [11, 12, 13], whereby individuals have increased long-term immunity to the first strain they were exposed to during their lifetime. Recent evidence suggests that there may be some level of immunity existing in populations even to novel influenza strains with pandemic potential [14, 15]. The presence of antibodies in an individual, particularly at low levels, may not prevent reinfection but may reduce the chance of infection when challenged or lead to a milder or asymptomatic illness [16, 17]. Further, many historical studies have relied on serological data as a means of determining if influenza infection occurred (e.g. [18, 19, 20]). However, the results of serological studies can vary substantially based on the criteria used [21], and produce estimates that are vastly different from active surveillance of symptomatic cases (e.g., [22]), and the relationships between antibodies and immunity is not completely understood [23].

Estimates for *R*_*e*_ are often between 1.0–2.0 (e.g., see [24]), however in rare cases estimates of *R*_0_ have been as high as 22.7 [25, 26]. While these most extreme values may be highly unlikely, estimates in the intermediate range are plausible when associated with reduced population susceptibility. Assumptions around parameter values can propagate through a modelling analysis, and impact on how we understand and characterise epidemic dynamics, e.g., when estimating the final size of a seasonal epidemic, or when calculating optimal vaccination policy. As such, careful analysis of high-quality data, estimating both population susceptibility and *R*_0_, is critical to inform present and future modeling efforts.

**Fig 1:**
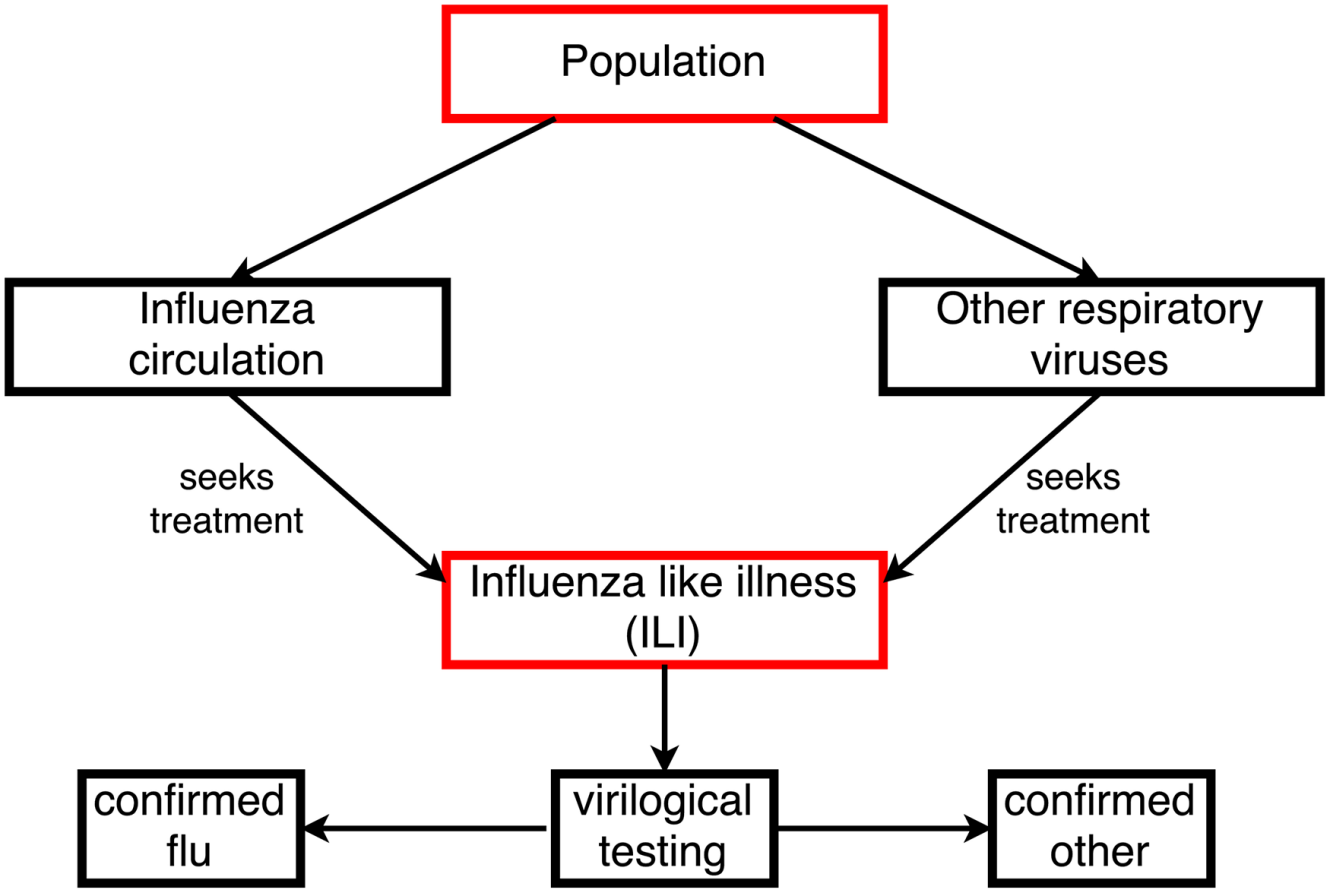
Schematic of influenza-like illness (ILI). Individuals with influenza or other respiratory viruses likely seek treatment at different, unknown rates, and as such ILI counts include an uncertain mixture of diseases. Virological testing is uncommon outside high risk groups, but when performed it is possible to use the resulting confirmed influenza data to model the population level circulation of influenza, specifically. Red quantities are those with known/observed values. Note that some ILI samples sent for testing return with as positive to none of the tested diseases, suggesting that their ILI symptoms may have some other cause.

**Fig 2:**
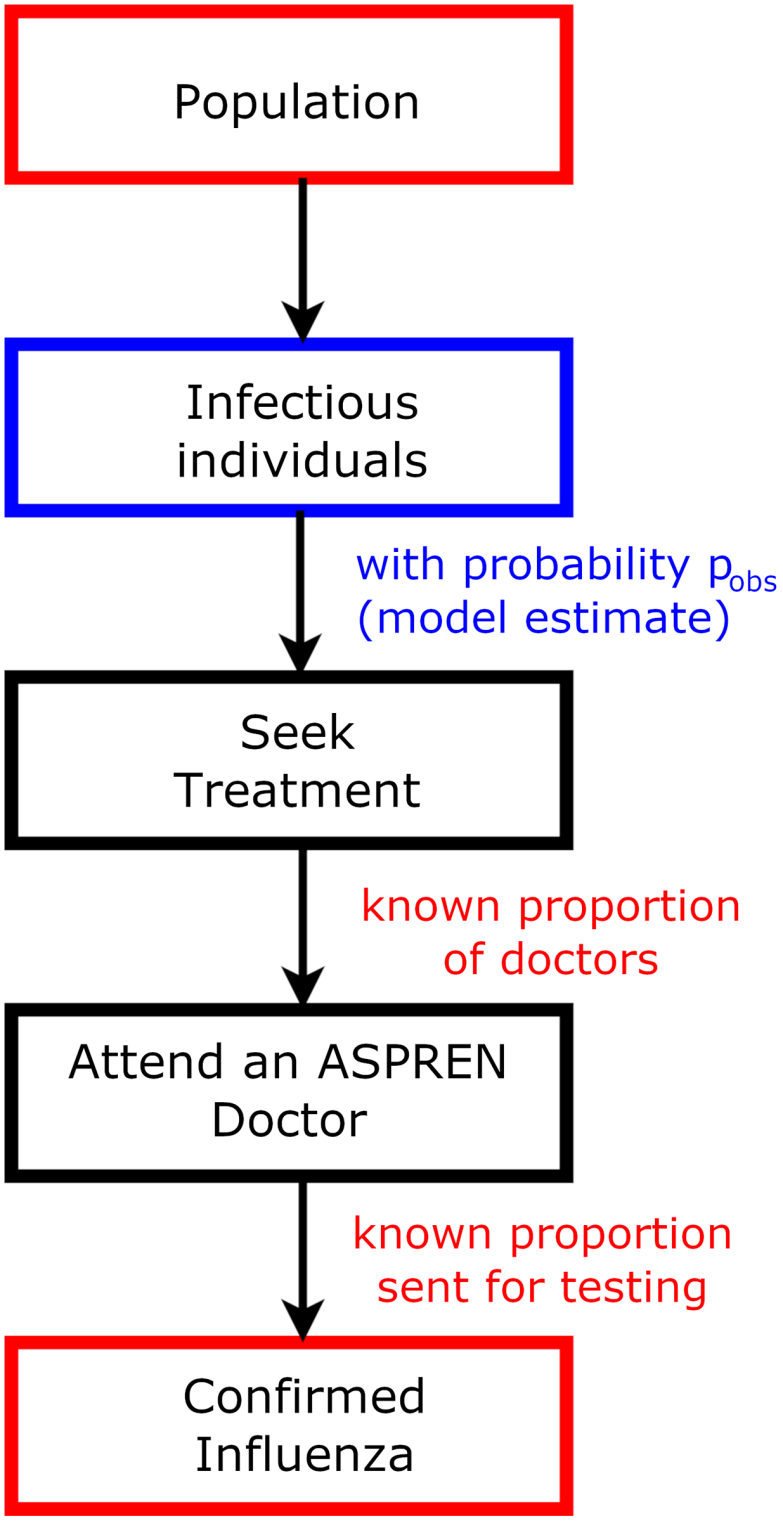
Hierarchical observation process in the presence of confirmed influenza data and known denominators. This structure allows us to directly model influenza without the other respiratory viruses that make up ILI. Red quantities are those with known/observed values, and blue quantities need to be estimated in a model.

Effective modelling of influenza dynamics is further complicated by the paucity and quality of available data. Influenza is difficult to identify clinically; patients presenting with influenza-like symptoms may have influenza (Fig 1), but they may also have some other respiratory virus, such as human rhinovirus, or respiratory syncytial virus [27]. To determine the actual cause of these symptoms requires a (relatively expensive) test, which may be appropriate for an at-risk individual presenting serious symptoms at a hospital but is unlikely to be necessary for an otherwise healthy individual in the 15–45 age group (for example). In addition, patients may be asymptomatic (while potentially still able to infect others), or those with mild symptoms may choose not to seek medical treatment at all. So, confirmed cases are likely to be only a subset of actual cases, with poor “denominator data”. The hierarchical observation process that links population level epidemic dynamics to observed confirmed cases (Fig 2) may make identifiability challenging and can in some cases introduce bias to estimates [28, 29, 30].

In this study, we used a new, high-quality dataset along with modern Bayesian parameter estimation methods to investigate seasonal influenza dynamics in New South Wales, Australia, from 2011–2016. We used Polymerase Chain Reaction (PCR) confirmed influenza infections from the Australian Sentinel Practices Research Network (ASPREN; [31]) to fit models. Within these data, approximately 0.5% of general practitioners (GPs; known as family physicians in North America) within Australia reported each Influenza-like illness (ILI) case that they observed, along with the number of consultations they performed, and submitted swab samples for c. 20% of these ILI cases for virological testing via PCR. These data were available weekly. Knowing the numbers of doctors, consultations, ILI observed, and samples tested provides critical and unparalleled denominator data against which to assess the hierarchical observation process between population-level influenza dynamics and the observed confirmed infections. We fit model parameters using approximate Bayesian computation (ABC), assuming the same strain-specific parameters for seasons in which the same predominant strain circulated. By performing this analysis using high-quality confirmed influenza data (including denominators), state of the art parameter estimation techniques, and minimal assumptions, we are able to characterize the epidemiology of influenza within populations. In doing so, we aim to both progress the science of disease modeling, and produce new epidemiological knowledge around population-level immunity to seasonal influenza.

## Model

**Fig 3:**
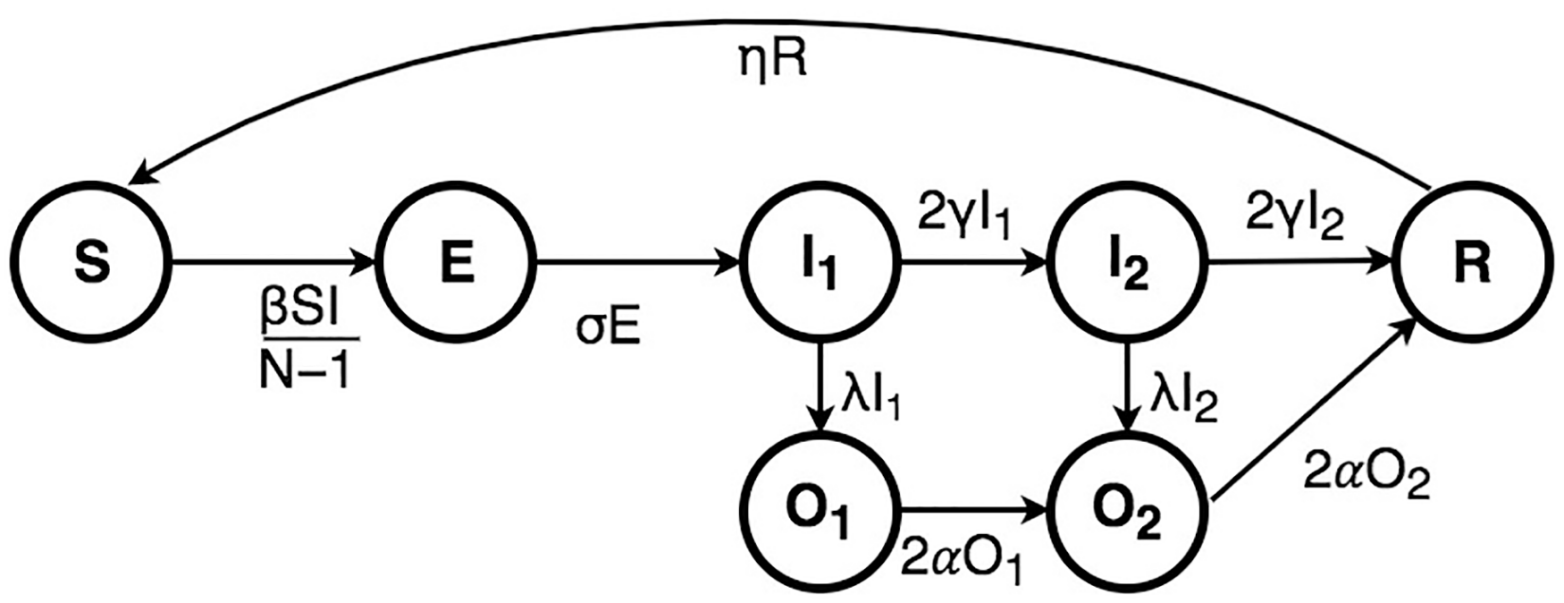
Model schematic. Individuals progress from a susceptible (S) class through exposed (E), infectious (I), and recovered (R). While infectious, individuals may choose to go to the doctor, at which case they move into an observed (O) class.

We chose to use an SEIRS-type model, with an Erlang-2 infectious period and exponential exposed and immune times, to model seasonal influenza (Fig 3), which is consistent with known influenza biology. In this form of model, we track the number of individuals in the population who are susceptible (S), exposed but not yet infectious (E), infectious (I), or recovered and immune from reinfection (R) at any time. We include an observed class (O) to allow individuals to choose to seek treatment, or not. Note we allow immunity to wane, so that individuals return from the recovered class to the susceptible class, and include external importation of cases at a low rate. Transition events are stochastic, with rates that depend on the number of individuals in each class, and fitted parameters that relate to the epidemiology of the disease. We fit the initial proportion of individuals that are susceptible to the strain circulating in the given season as a parameter, and model seasons that had the same predominant circulating strain together, i.e., 2011 and 2013 both had predominantly H1N1pdm09 circulating, so we fit these seasons to have the same epidemic parameters, but each with a separate initial population susceptibility. The presence of an exposed class in the chosen model indicates that there is some lag between when an individual is exposed to the disease, and when they become infectious. We assume that they are not symptomatic during this exposed period. This is consistent with many existing models and known influenza epidemiology [32].

By using models aggregated at a state level, without any age- or spatial- structuring, we make a number of simplifying assumptions. Specifically, we lose the ability to model heterogeneity in mixing and thus infection rates, vaccination coverage, immunity, or case ascertainment. We know that these factors should, in reality, be heterogeneous, as there is strong evidence for variations based on (in particular) age structure [33, 34]. However, the data we have access to are unfortunately too sparse to effectively parametrize models at this fine level of detail. In some cases, this may introduce some degree of bias. For example, newborn children entering the population are necessarily susceptible, and by not tracking demographics we may overestimate the rate at which immunity wanes (i.e., underestimate the duration of immunity), which could potentially have some impact on estimates of other parameters, including *R*_0_.

## Results

### Posterior distributions

Having chosen the SEIRS-type model, we used ABC to estimate posterior distributions for model parameters, and to evaluate the impact of these fitted model parameters on underlying epidemic dynamics at the population level. We parameterized transmission based on physical quantities, including the latent, infectious, and immune durations, the probability of an infectious person seeking treatment over their infectious period, the basic reproduction number, *R*_0_, and the proportion of individuals susceptible to the circulating strain at the beginning of each season. *R*_0_ was assumed to have seasonal forcing, for which we used variations in temperature (*T*) from the annual mean ($\bar{T}$), i.e., $R_{0}={\bar{R}}_{0}+a\left(T-\bar{T}\right)$, with ${\bar{R}}_{0}$ and *a* being estimated parameters. Alternate seasonal forcing terms were tested (e.g., based on specific humidity with the same form as Yang *et al*. [35]), however these produced similar quality results while requiring more parameters, and were harder to interpret. We therefore chose to use temperature-based forcing for simplicity. Results were broadly similar between strains, so in the text we report primarily the H1N1pdm09 results (circulating in 2011 & 2013); a summary of H3N2 (2014 & 2016) appears in Table 1, and figures appear in S1 Text.

**Table 1:** Marginal posterior parameter estimates—H1N1pdm09 seasons.

parameter	posterior			prior	
	median	mode	90% credible interval	distribution	90% credible interval
${\bar{R}}_{0}$	4.73	2.03	(1.7,15.8)	Exponential(1/22.7) (truncated at 22.7)	(0.722,20.804)
*R*_0_ seasonality term (*a*)	-0.0617	-0.0168	(-0.30233, -0.00702)	- Exponential(1/0.2)	(-0.603, -0.0104)
latent duration (days)	1.26	2.78e-05	(0.0961, 4.6222)	Exponential(1/1)	(0.0505, 2.9773)
infection duration (days)	3.97	3.23	(0.612, 10.854)	Exponential(1/2)	(0.102, 5.978)
immune duration (years)	24	11.8	(3.64, 84.86)	Exponential(1/27.1)	(1.4, 82.0)
Probability of seeking treatment	0.237	0.0843	(0.0505, 0.8081)	Uniform(0,1)	(0.05, 0.95)
initial susceptibility 2011	0.224	0.0799	(0.0603, 0.6436)	Uniform(0,0.75)	(0.0375, 0.7125)
initial susceptibility 2013	0.188	0.0762	(0.0395, 0.6015)	Uniform(0,0.75)	(0.0375, 0.7125)

#### Correlation from posterior distributions: *R*_0_, susceptibility, and observation

**Fig 4:**
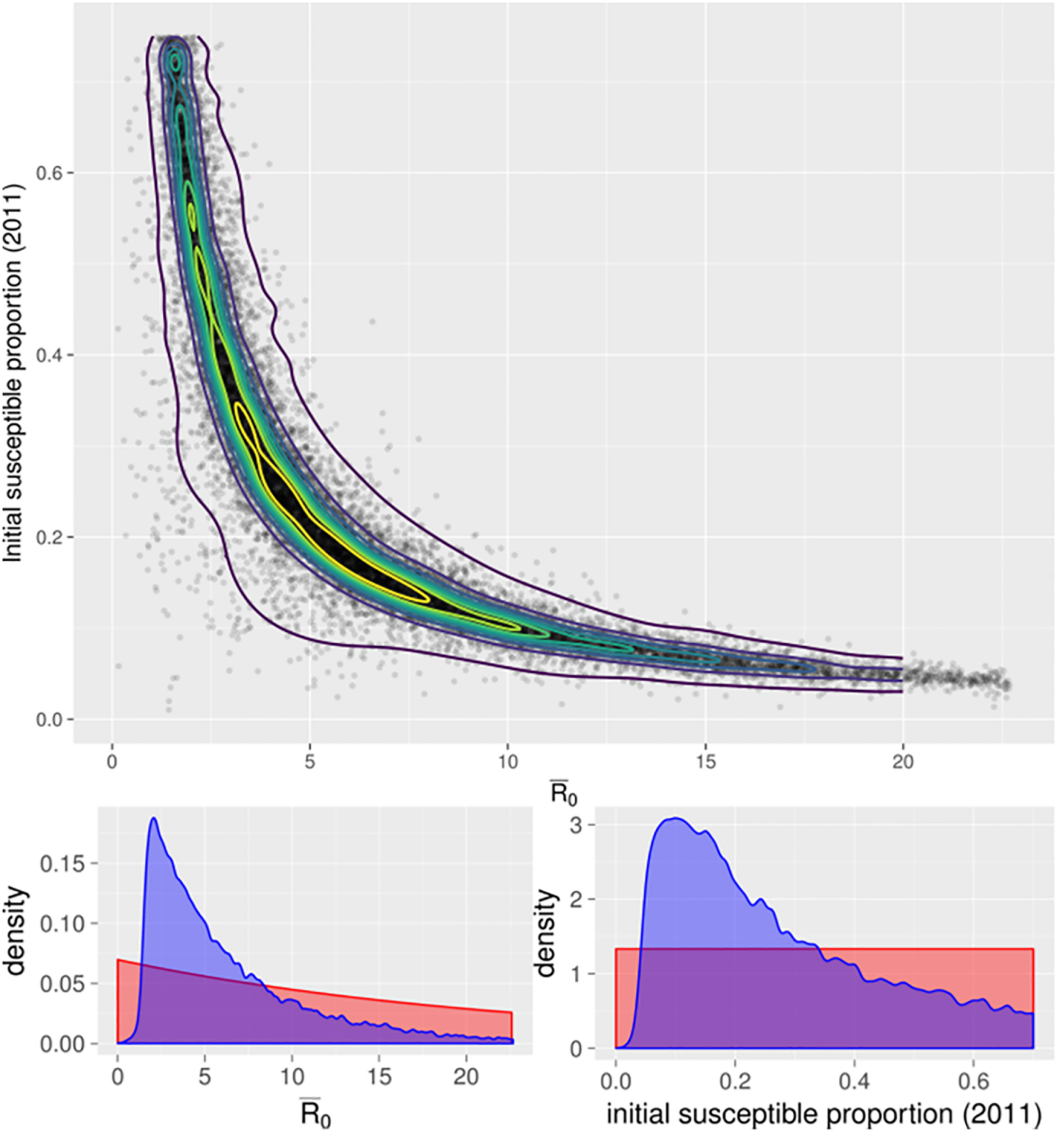
(above) Bivariate posterior distribution of ${\bar{R}}_{0}$ and initial susceptible proportion in 2011 for H1N1pdm09. Points indicate accepted ABC parameter sets. Contours indicate posterior credible intervals, such that each contour contains deciles of kernel-smoothed posterior probability density. (below) Marginal posterior kernel density estimate (blue) and prior distribution (red) for ${\bar{R}}_{0}$ (left), and initial susceptible proportion in 2011 (right) for H1N1pdm09. Note the difference between high-density regions in the bivariate plot vs the marginals.

In the posterior distribution, small *R*_0_ values were associated with large initial susceptible populations, and vice versa, such that the product of mean *R*_0_ and initial susceptibility was approximately 1.0 (Fig 4). The correlation between $1/{\bar{R}}_{0}$ and initial population susceptibility was 0.92. The bivariate posterior density covered the full range of this parameter space with the (bivariate) posterior mode occurring at ${\bar{R}}_{0}=5.82$ and initial population susceptibility of 0.179 (Fig 4). However, note that there was high posterior density across a broad range of parameter values, with the 80% quantile including values of ${\bar{R}}_{0}$ across the range 2–10. In contrast, the marginal posterior estimates of these parameters had modes that varied substantially from the bivariate distribution; with the maximum posterior estimate of ${\bar{R}}_{0}$ at 2.03 (90% credible interval 1.7–15.8), and for initial susceptibility, 0.08 (90% credible interval 0.06–0.64) for H1N1pdm09 seasons. This highlights the importance of considering relationships between parameters and identifiability issues, rather than relying solely on marginal distributions when parameters are not independent.

There is a body of literature suggesting that *R*_0_ for influenza is in the 1.0–2.0 range [24]. However, based on these data we have observed most of the posterior density falls outside this range when a broad prior is used (Fig 4); specifically, the lower limit of the 90% credible interval for ${\bar{R}}_{0}$ is 1.7, with only 10.6% of accepted parameter sets (2099 of 19772) below 2.0.

The median posterior density for the initial susceptible proportion was 0.224 in 2011 and 0.188 in 2013, suggesting that most individuals are unlikely to be susceptible to the circulating strain of influenza each year, even when that strain was not the primary circulating strain in the previous season (however, note again that there was substantial uncertainty in estimates of initial susceptibility, and it was related to ${\bar{R}}_{0}$ in the posterior). We also fit a parameter for mean immune duration, however this parameter was not identifiable given that seasonal fits were not consecutive, and other strains proliferated in the intervening seasons with complex and uncertain effects on immunity.

**Fig 5:**
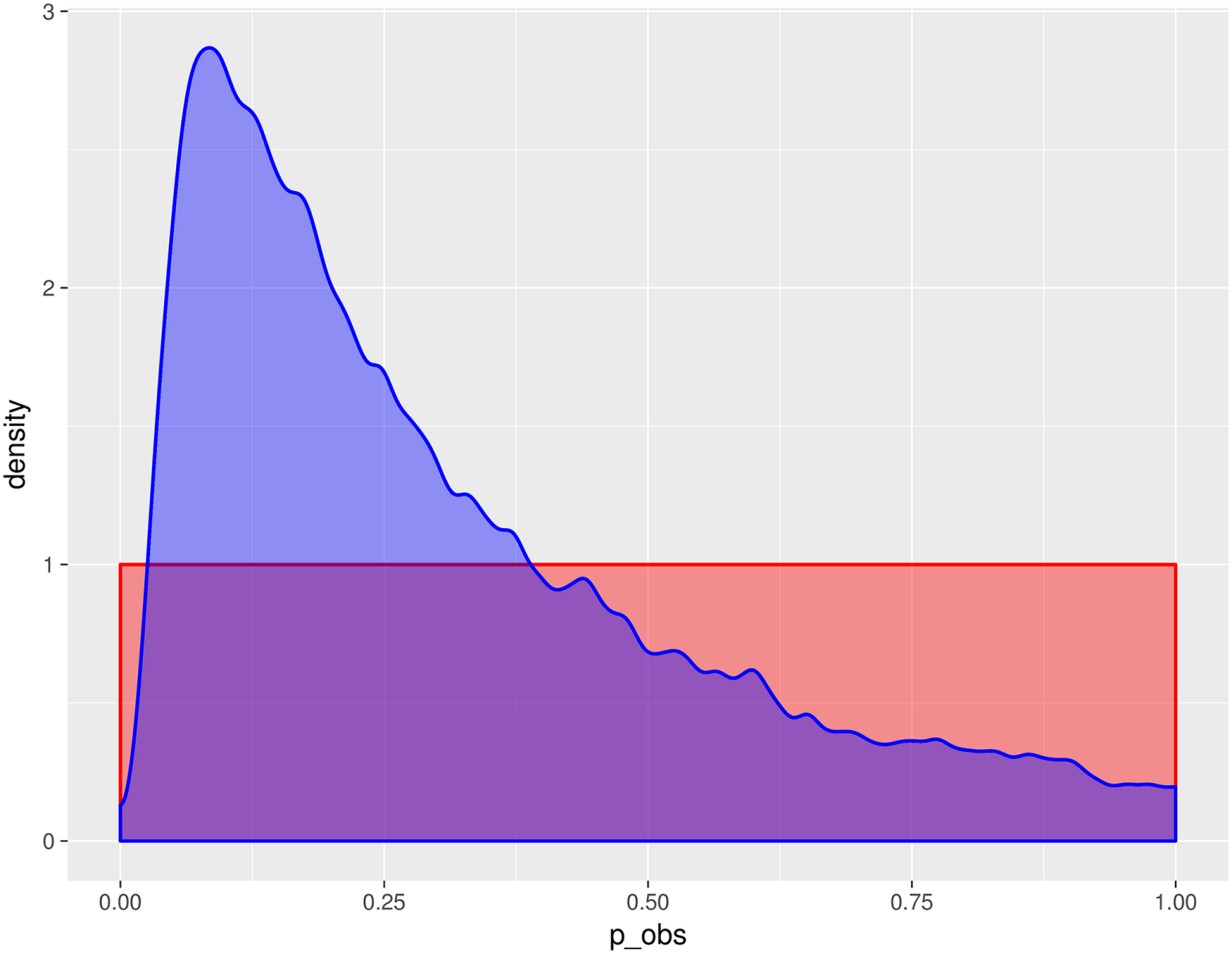
Marginal posterior kernel density estimate (blue) and prior distribution (red) for *p*_obs_, for H1N1pdm09.

**Fig 6:**
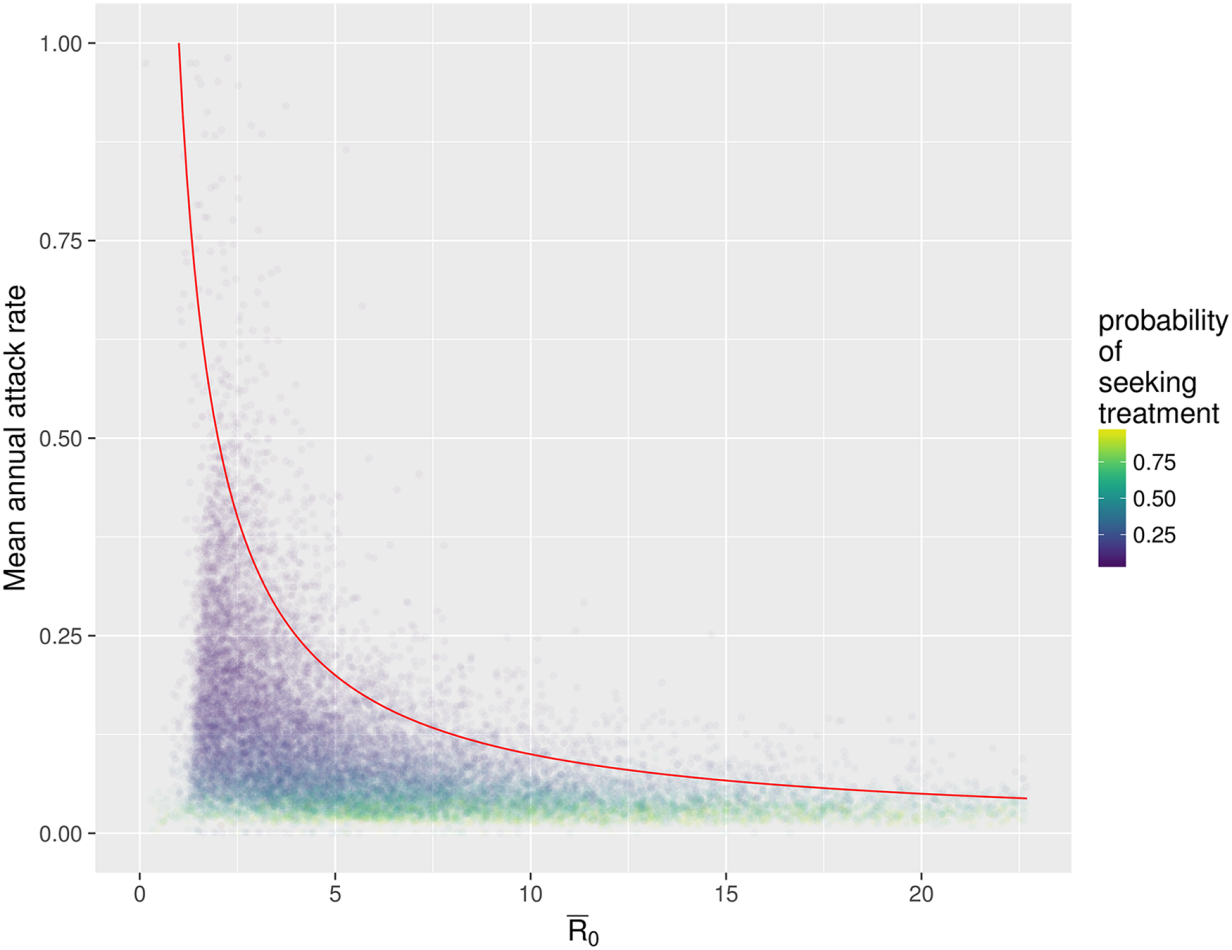
The relationship between ${\bar{R}}_{0}$ and the population level attack rate of simulated realisations of the process, across 2011 and 2013 H1N1pdm09. For each accepted particle, the process was simulated again, and the total number of infected individuals from these new realisations was recorded, and converted into an annual attack rate (by dividing by the total population, and by 2 as it is the average over two seasons). Point colour indicates the probability of an individual seeking treatment; larger attack rates correspond to smaller treatment probabilities given that they all fit the same data. The red line shows where the product of ${\bar{R}}_{0}$ and the attack rate would be equal to one; this is the line around which the majority of posterior density for initial population susceptibility values was situated (i.e., the density in Fig 4). This can be interpreted as indicating that, for points under this line, not all susceptible individuals became infected during the season.

The probability that an individual seeks treatment, *p*_obs_, is also critical to understanding influenza dynamics and surveillance. The median posterior estimate for *p*_obs_ for H1N1pdm09 seasons was 0.237 (Fig 5), however the 90% credible interval was quite wide (0.0505—0.8081), suggesting that this parameter remains challenging to identify given these data. The probability of seeking treatment was also related to ${\bar{R}}_{0}$ and initial susceptibility. Specifically, those accepted particles with high initial susceptibility and lower ${\bar{R}}_{0}$ also had smaller observation probabilities (Fig 6), and, consequently, larger (population-level) epidemics.

When simulating from the posterior distributions (Fig 6), the range of parameter estimates accepted in the posterior induce a diversity of associated epidemic characteristics. Specifically, population epidemic metrics such as the attack rate (total proportion of the population infected over a season), and the proportion of the population susceptible at the beginning of a season, are related to *R*_0_ and the probability an individual seeks treatment (Fig 6). The majority of posterior probability mass is around the region in which 10-30% of individuals in the population are susceptible at the beginning of a season, and 75% of accepted particles had an attack rate below 14.1% (less than 970,000 infections occur per year). An attack rate in this region does align with previous estimates in other studies [36]. However, parameter sets are also accepted in regions of parameter space resulting in larger proportions of susceptible individuals at the beginning of the season, associated with larger epidemics, and smaller probabilities that each infectious individual seeks primary care. We note that these estimates more closely resemble modelling results such as those of [13], who estimated susceptibility and attack rate using a multistrain model and assuming *R*_0_ = 3.0. Only c. 15.4% of accepted parameter sets had ${\bar{R}}_{0}$ in the range 2.5 < *R*_0_ < 3.5, and simulations from these parameter sets resulted in c. 37.2% of individuals being susceptible at the start of a season, and c. 880,000 annual infections (c. 12.7% attack rate), vs 22.4% susceptibility and c. 7.7% attack rate (medians) for the full posterior.

#### Latent and infectious periods

The median posterior density for the mean infectious duration was 3.97 days, and for the latent duration was 1.26 days. These estimates align well with historical data on influenza epidemiology [32]. However, for each of these parameters, the posterior credible intervals were wide, and closely resembled the prior. Given the weekly resolution of the data it is unlikely that these parameters could be identified more precisely.

### Variation between seasons and strains

Initial susceptibility values in seasons of the same strain were highly correlated (e.g. correlation coefficient 0.90 between initial susceptibility in 2011 and 2013). The posterior densities had similar forms between strains, however the H3N2 seasons (2014 & 2016) had slightly lower median ${\bar{R}}_{0}$, associated with slightly higher initial susceptibility (Tables 1 & 2). For H3N2 seasons, the maximum bivariate posterior density occurred at ${\bar{R}}_{0}=4.71$ and initial susceptibility 0.22 (in 2014) (S1 Text).

**Table 2:** Marginal posterior parameter estimates—H3N2 seasons.

parameter	posterior			prior	
	median	mode	90% credible interval	distribution	90% credible interval
${\bar{R}}_{0}$	3.93	2.16	(1.65, 13.03)	Exponential(1/22.7) (truncated at 22.7)	(0.722, 20.804)
*R*_0_ seasonality term (*a*)	-0.0973	-0.0512	(-0.3539, -0.0189)	- Exponential(1/0.2)	(-0.603, -0.0104)
latent duration (days)	1.32	6.88e-05	(0.0953, 4.6134)	Exponential(1/1)	(0.0505, 2.9773)
infection duration (days)	3.93	2.88	(0.511, 10.775)	Exponential(1/2)	(0.102, 5.978)
immune duration (years)	22.6	8.51	(3.52, 86.72)	Exponential(1/27.1)	(1.4, 82.0)
Probability of seeking treatment	0.202	0.0893	(0.0518, 0.7238)	Uniform(0,1)	(0.05, 0.95)
initial susceptibility 2014	0.275	0.138	(0.0692, 0.6657)	Uniform(0,0.75)	(0.0375, 0.7125)
initial susceptibility 2016	0.26	0.134	(0.0663, 0.6336)	Uniform(0,0.75)	(0.0375, 0.7125)

## Discussion

An understanding of seasonal influenza transmission is of critical public health concern, however effective inference can be constrained by the availability of high-quality data. In this study, we have used confirmed, multi-year virological data from a primary care surveillance network to perform model parameterisation for seasonal influenza in New South Wales, Australia. Making minimal assumptions around parameter values, we observe strong posterior relationships between *R*_0_ and population susceptibility, with higher *R*_0_ values corresponding to lower levels of susceptibility to the circulating strain. These results emphasise the challenges of identifying parameters around susceptibility, transmission, and observation, and highlight the substantial uncertainty that remains in these areas. We encourage caution when making modeling assumptions, choosing priors, and interpreting results, particularly when parameters are related.

The point that *R*_0_ is not clearly identified in influenza data has been made previously [7, 8], observing that many studies report *R*_0_ while actually estimating *R*_*e*_ = *R*_0_*S*_0_, where *S*_0_ is the proportion of the population that is initially susceptible. Our results provide an additional line of evidence to reinforce this idea, with the majority of accepted parameter sets having parameters and model states such that *R*_0_ times the proportion of individuals that are susceptible at the start of a season lies between 1 and 2, whereas actual *R*_0_ values are generally much higher. The maximum posterior estimates when both *R*_0_ and susceptibility were considered had ${\bar{R}}_{0}=5.81$ (for H1N1pdm2009 seasons), however there was substantial uncertainty in this estimate, with high posterior density across the range 2–10. This is consistent with previously calculated *R*_0_ values for historic pandemic influenza seasons or within populations that were likely to have high levels of initial susceptibility, such as on the island of Tristan da Cunha in 1971, with *R*_0_ in the range 3.73–10.69 [37]. The highest extreme of our posterior estimates for *R*_0_ exceed these estimates (in association with very low population susceptibility), and may be so high as to be implausible outside of unique outbreak scenarios. It is likely that with more data, posterior estimates would become more precise, however given the data available these extreme values cannot be discarded. We also emphasise that in epidemic studies marginal parameter estimates should be considered with caution, as maximum posterior density estimates from marginal distributions are very different to those from bivariate posterior distributions, given the strong nonlinear relationships between key parameters. This is illustrated clearly by comparing Figs 4 and 5.

While there may be cases where only the effective reproduction number is important, it is, in our view, generally inappropriate to model a seasonal virus that has underlying immunity within a population without specifically considering that immunity. Doing so could create bias in predictions or model outputs, and, in the worst case, detrimentally affect public health outcomes if or when incorrect parameter estimates are used to inform resource allocation or vaccination strategies. For example, it may be more effective to target vaccination resources at individuals or households (or geopolitical regions) with higher likelihoods of susceptibility based on infection history in previous years. This likely differs from the optimal strategies if underlying immunity is ignored. Forecasting in the presence of mutation or antigenic drift is also of concern, with model assumptions likely to lead to vastly differing predictions particularly when made based on limited data at the start of a potential pandemic. Moreover, confusion over estimates may lead to problems when making comparisons between diseases. In every case, researchers should critically consider the modeling assumptions being made and the impact that they may have on parameter estimates and subsequent analyses.

Observing potentially high transmissibility alongside lower susceptibility for seasonal influenza supports the idea that population susceptibility, rather than increased transmission, is the primary factor differentiating influenza strains with pandemic potential at a particular time from strains exhibiting routine seasonal circulation. The H1N1pdm09 strain that emerged and caused a pandemic in 2009 provides a specific example: when we fit parameters to this same strain in 2011 and 2013, it had relatively low levels of population susceptibility, and therefore routine, seasonal epidemics. It is likely that the difference between the circulation of the strain in 2009 and later years is due to differences in population susceptibility: because there were no similar strains circulating prior to 2009, a large proportion of the population (and specifically, anyone born after the most recent pandemic of a similar strain, in the late 1970s) was effectively naive, or only protected by cross-immunity. A confounding observation is that many estimates of *R*_*e*_ during pandemic seasons have fallen in the range 1.0-2.0 [24]; whereas we may expect these estimates to by higher given increased population susceptibility. There are myriad reasons which could contribute to lower than expected estimates of transmissibility in a pandemic. The most critical factor is that, while population susceptibility is increased relative to seasonal circulation, there remains some level of immunity within populations, particularly in older age cohorts, either due to previous exposure to similar strains or cross-immunity with other strains [8, 15, 38, 39]. Additionally, studies which estimated *R*_*e*_ for the 2009 pandemic highlighted possible impacts of factors including: public-health containment measures or changes in public behaviour due to pandemic awareness [38, 40, 41, 42]; outbreak timing coincident with school vacations [40, 43]; climatic conditions (with the initial outbreak occurring during summer in the Northern Hemisphere [42, 43]); or challenges in data collection (including sampling associated specifically with public-health programs, and potential variation in case ascertainment across age groups [39, 40]). It is also important to note that novel strains (e.g., transmitted from animal hosts) require the capacity to spread in humans, in addition to novelty and sufficient population susceptibility, before they can induce a pandemic [44].

We acknowledge that our results, particularly surrounding high *R*_0_ estimates in the bivariate posterior distribution, are provocative, and may challenge conventional wisdom. While we have endeavoured to take a principled approach that makes as few assumptions as possible, there are aspects of the study with the potential to introduce bias, and there remains substantial challenges in identifiability between parameters, particularly *R*_0_, the proportion of individuals that are susceptible, and the probability an individual seeks treatment. Specific modelling choices that could contribute to higher-than-expected *R*_0_ values are discussed in subsequent paragraphs. These include:

- Choice of priors, which inevitably impacts the resulting posterior distributions; restricting priors to lower *R*_0_ values would necessarily result in lower posterior estimates of *R*_0_.
- Modelling all influenza circulation within a season as a single strain (i.e., the primary strain circulating in that season), when in practice there are low levels of other strains circulating.
- Constant population size, and thus not tracking population turnover within a season.
- Homogeneous mixing and a single age class (rather than an age-structured population), given that in practice parameters including transmissibility, susceptibility, and treatment seeking behaviour vary with age in complex ways.

It is difficult to be certain of the impact of particular assumptions on parameter estimates, given the complex interactions between parameters. It is possible that, combined, these modelling assumptions could have contributed to higher-than-expected posterior estimates of *R*_0_. Ultimately, there remains substantial uncertainty around parameter estimates, with wide credible intervals and correlated posterior distributions consistent with the challenges of identifiability for *R*_0_, susceptibility, and treatment seeking behaviour. The present study does not claim to provide a definitive answer, rather it should inspire further research with high-quality datasets and careful analysis that at least considers a full range of plausible priors, and that considers interactions between parameters rather than marginal estimates.

It is important to emphasise that throughout this study we chose to use general, uninformative priors, in an effort to avoid imposing any strong assumptions around parameter values. A reasonable alternative approach to this would be to use information from the literature to impose informative priors, such as by reducing the prior density for larger values of *R*_0_, or making assumptions around population susceptibility. It is possible that informative priors could shift the resulting posterior distributions, and even impact the way that the study is interpreted. We emphasise the importance of carefully considering the priors used for this type of modelling study, and in interrogating how these choices might impact modelling outcomes.

A key limitation of this study is that we considered each of the study years to have only a single circulating influenza strain, which disregards the low levels of circulation of other strains that did occur. The impact of the circulation of these other strains, even at low levels, could potentially bias parameter estimates. Constructing distinct models for the different strains means that we could not use these models to understand cross-immunity between strains. Models that consider multiple strains and their interactions, or asymptomatic infections, would be ideal, but parametrizing these more realistic models is likely to be very challenging without both a better epidemiological understanding of how immunity and cross-immunity between strains works, and high resolution data including these strains. A further critical limitation of this work is that we did not consider population turnover or age structure. Age structure plays a key role in immunity, treatment-seeking behaviour, and transmission between individuals. By not including age structure, we necessarily assume that individuals mix homogenenously with age, that immunity is uniform across ages and wanes consistently, and that vaccination coverage is uniform across age groups: all of which are known to be incorrect assumptions. Future work should seek to include age structure in models such as this, while seeking to ensure that the available data and computation resources are sufficient to effectively fit additional parameters. It is likely that to produce detailed models with cross-immunity and age structure, a detailed longitudinal cohort study would be required, including regular sampling of multiple types (e.g., serology and virology) and potentially movement or contact tracing. Existing studies such as the Fluscape cohort study [45] will provide a starting point, however more regular sampling regimes may be necessary to elucidate the most complex dynamics.

While high-quality data and modern parametrization methods are helpful, there are limits to what may be reasonably determined using highly filtered weekly data. We are not able to effectively discriminate infectious durations, nor latent periods, and credible intervals on many parameters (e.g., the probability of seeking treatment) were quite broad. It is conceivable that this may be possible with daily data, additional testing, or by incorporating other data sources.

It is important to emphasise that the modelling in this study directly considers confirmed influenza. Such data are not typically directly available, particularly with denominator data. Were it more common to report negative test results (or, test results positive for other respiratory viruses), then alternate data sources of confirmed influenza, such as national notifications databases, would be more valuable and provide higher resolution with which to investigate seasonal influenza.

### Conclusion

Seasonal influenza can be a challenging process to characterize given the complex nature of immunity and the inability to discriminate influenza from other respiratory viruses without testing. In this study, we used confirmed virological influenza data, with known denominators, to ensure that the model is specifically based on true influenza cases and the hierarchical observation process is accurate, and modern Bayesian inference techniques, with uninformative, unbounded priors, to ensure that assumptions around these priors did not impact on the resulting parameters estimates. We identified strong posterior relationships between *R*_0_, population susceptibility, and the probability an infected individual seeks treatment. The bivariate posterior distribution had maximum density where relatively high *R*_0_ values correspond with low levels of population susceptibility (while the effective reproduction number *R*_*e*_ remains within the expected range). This was in sharp contrast to the values that would be obtained by considering only the marginal posterior distribution of each parameter. This highlights the importance of carefully considering the challenges of identifiability in parameter estimates, and in particular the importance of considering immunity alongside transmissibility in modelling studies. While there remains uncertainty around parameter estimates, we encourage researchers to consider carefully the challenges of identifying related parameters, and the impact that strong prior and modeling assumptions can have on parameter estimates.

## Methods

Data were obtained as part of the ASPREN project ([31]; https://aspren.dmac.adelaide.edu.au/), a database of general practitioners (GPs) distributed throughout Australia. The target coverage is approximately one GP per 200,000 people in metropolitan areas, and one GP per 50,000 people in regional areas. This database is continually updated and requires the voluntary participation of GPs, so there is variation between years and states. All GPs in the database reported to ASPREN the number of consultations they performed each week, along with every case of influenza-like-illness (ILI) that they observed. GPs were asked to take swab samples from a proportion of those patients presenting ILI symptoms—25% from 2012–2014, 20% in 2015–2016. In practice, not all GPs took samples, and the proportion of patients for which samples were taken varied substantially, however we use exact denominators accounting for the testing doctors and the number of ILI patients they observed. These swab samples were sent for PCR testing, resulting in respiratory virus virology data for each sample. In this study, we used those data from New South Wales, as this is the most populous state in Australia and has the most confirmed influenza cases.

Because influenza has multiple strains, interactions between those strains are complex, and immunity from one strain may not confer protection to a different strain, we chose to model seasons with different strains separately. Specifically, the predominant strain in 2011 and 2013 was H1N1pdm2009, and the predominant strain in 2014 and 2016 was H3N2, so we performed model fitting separately on these two datasets. In doing so, we assume that epidemic parameters (e.g., *R*_0_) were consistent across seasons for a single strain, and we explicitly fit the proportion of the population susceptible and number infected at the start of each season. In 2012 and 2015 there were multiple strains with substantial circulation, and so we chose not to fit these seasons rather than making strong assumptions around interactions between strains.

Historical climate data were obtained from the Australian Government Bureau of Meteorology (www.bom.gov.au/climate/data), at station 066062 (Sydney—Observatory Hill). We used climate data from Sydney, the state capital, as both the majority of the population and the majority of GPs in the study were located here. Climate data consisted of observations of temperature, precipitation, and absolute humidity, taken every three hours (i.e., 8 times per day), continuously from 1955–present. Note that three-hourly observations creates a cyclic temperature pattern with both daily and annual periods. So as to avoid having unrealistic diurnal dynamics, we used the average daily temperature rather than the three-hourly observations themselves. We used a fixed, total population size of 6.9 million, based on 2011 Australian Bureau of Statistics estimates. While the population of New South Wales is increasing over time, including demography would add considerable complexity, which we wished to avoid.

### Hierarchical observation process

The critical concern when using these data is an understanding of how underlying population-wide influenza dynamics translate to observed, confirmed influenza cases in the available dataset. We propose that filtering occurs at three levels:

- Not all influenza cases are identical in morbidity, and given mild or asymptomatic cases patients may or may not choose to consult a GP. We assume that each infectious individual has some (unknown, independent and identically distributed) probability of consulting a GP.
- Only a small proportion of GPs are part of the ASPREN network, and the proportion that are has varied over time. We then assume that an infectious patient who attends a GP has some probability (fixed, estimated from data, varying annually) of that GP being part of the ASPREN network.
- ASPREN doctors are instructed to send a proportion of their ILI cases for swab testing. This target proportion has varied over time, and the proportion of cases that doctors actually send for testing has varied significantly beyond that. We assume that each infectious patient who attends an ASPREN GP has some probability (fixed, estimated from data, varying weekly) of being sent for testing.

There may well be variation from these fixed parameters in practice. However, if all parameters were allowed to vary freely then it would not be possible to identify them, as only the result of all three levels of filtering is observed.

### Models

We considered a continuous-time stochastic epidemic model, with an SEIRS structure, with an observation class and Erlang-2 sojurn times in the infectious class (modelled by splitting these classes into two consecutive compartments) (Fig 3). This process was approximated in discrete time with 8 timesteps per day. So as to avoid diurnal dynamics with transmission rates varying within days and being maximised in the early hours of the morning, we used the daily mean temperature at each timestep. We also allow external importation of cases, at a per-susceptible rate *ξ* (constant through time) which we fit as part of the model. Note that because of the structure of the model, the mean infectious time of an individual is 2/(2*γ* + λ) (see S1 Text). The model was parameterized based on physical epidemiological quantities, which were then transformed into model-based rates, which appear in Table 3.

**Table 3:** Relationship between fitted epidemic quantities and transformed model parameters. Multiplying by 8 in these transformations is to account for using 8 timesteps per day.

fitted epidemic quantity	transformed model parameter
infectious duration *D*_inf_	recovery rate parameter $\gamma =\frac{\sqrt{1-p_{\text{obs}}}}{8D_{\text{inf}}}$
probability of seeking treatment *p*_obs_	treatment-seeking rate parameter $\lambda =\frac{2-2\sqrt{1-p_{\text{obs}}}}{8D_{\text{inf}}}$ *α* = 3*γ* + λ
mean basic reproduction number ${\bar{R}}_{0}$ seasonality term *a*	$R_{0}={\bar{R}}_{0}+a\left(T-\bar{T}\right)$, infection rate *β* = *R*_0_(2*γ* + λ)/2
rate of external importation *ξ*	not transformed
immune duration	waning immunity rate *η* = 1/(8 × immune duration)
latent duration	rate of becoming infectious *σ* = 1/(8 × latent period)
proportion susceptible at season start $S_{0}^{year}$	not transformed
infected individuals at season start $I_{0}^{year}$	not transformed

Given these transformed parameters, transitions for the SEIR model are as appears in Table 4, using the increments that appear in Table 5.

**Table 4:** Model transitions.

transitions
*S*_*t*+1_ = *S*_*t*_ + Δ*R*_*t*_ − Δ*S*_*t*_
*E*_*t*+1_ = *E*_*t*_ + Δ*S*_*t*_ − Δ*E*_*t*_
$I_{1,t+1}=I_{1,t}+\Delta E_{t}-\Delta I_{1,t}^{I}-\Delta I_{1,t}^{O}$
$I_{2,t+1}=I_{2,t}+\Delta I_{1,t}^{I}-\Delta I_{2,t}^{R}-\Delta I_{2,t}^{O}$
$O_{1,t+1}=O_{1,t}+\Delta I_{1,t}^{O}-\Delta O_{1,t}$
$O_{2,t+1}=O_{2,t}+\Delta I_{2,t}^{O}+\Delta O_{1,t}-\Delta O_{2,t}$
$R_{t+1}=R_{t}+\Delta I_{2,t}^{R}+\Delta O_{2,t}-\Delta R_{t}$

**Table 5:** Single-timestep increments for each model compartment.

increments
$\Delta S_{t}=\text{Binomial}\left(S_{t},1-\text{exp}\left(-\left(\beta \frac{\left(I_{1}+I_{2}+O_{1}+O_{2}\right)}{N-1}+\xi \right)\right)\right)$
Δ*E*_*t*_ = Binomial(*E*_*t*_, 1 − exp(−*σ*))
$\left(\Delta I_{1,t}^{I},\Delta I_{1,t}^{O}\right)=\text{Multinomial}(I_{1,t},\frac{2\gamma }{2\gamma +\lambda }\left(1-\text{exp}\left(-\left(2\gamma +\lambda \right)\right)\right),\frac{\lambda }{2\gamma +\lambda }\left(1-\text{exp}\left(-\left(2\gamma +\lambda \right)\right)\right))$
$\left(\Delta I_{2,t}^{R},\Delta I_{2,t}^{O}\right)=\text{Multinomial}(I_{2,t},\frac{2\gamma }{2\gamma +\lambda }\left(1-\text{exp}\left(-\left(2\gamma +\lambda \right)\right)\right),\frac{\lambda }{2\gamma +\lambda }\left(1-\text{exp}\left(-\left(2\gamma +\lambda \right)\right)\right))$
Δ*O*_1,*t*_ = Binomial(*O*_1,*t*_, 1 − exp(−2*α*))
Δ*O*_2,*t*_ = Binomial(*O*_2,*t*_, 1 − exp(−2*α*))
Δ*R*_*t*_ = Binomial(*R*_*t*_, 1 − exp(−*η*))

We chose this parameterisation so as to put priors on the physical quantities of interest. That is, priors were defined on *R*_0_, mean latent, infectious and immune durations, and the probability of observation over the infectious period, and then transformed to obtain epidemic model parameters such as *β* and *γ*. Initial conditions were fit for each season, so that susceptibility to the circulating strain in that season could be determined. Prior distributions for epidemic parameters appear in Table 1. Per-susceptible external importation of cases at a low rate was used to allow for the possibility of epidemic fade-out and reintroduction from elsewhere, and was assigned an exponential prior distribution with rate 10^9^ (approximately 3 individual case importations per year per million susceptible individuals). We did not reject simulation runs in which fade-out occurred, except to require circulation of influenza in any week in which there were influenza cases observed.

We chose to use exponential prior distributions so as to avoid putting maximum limits on the values these parameters may take, i.e., in the same sense that you would choose uniform priors to be uninformative, but with infinite positive support. Rates for the infectious and latent period exponential priors were chosen so that the means were within a reasonable range of influenza epidemiological parameters. The prior for ${\bar{R}}_{0}$ was initially chosen as Exponential with large mean so as to have a relatively flat density; we truncated this prior at the highest published estimate of *R*_0_ for influenza, 22.7 [25]. While arguments could be made for a more informative prior with further reduced density at higher *R*_0_ values, we chose to retain this relatively uninformative prior so as to minimise modelling assumptions. The probability that an individual chooses to seek treatment was assumed to be constant, both within and between seasons. In practice this may not be the case, particularly when a circulating strain is unusually transmissible, or produces stronger symptoms (in either case, potentially leading to greater media attention). However, the hierarchical observation structure means that we are able to capture some of this effect in the variation in testing probabilities, as we explicitly use the actual testing proportions given in our dataset, which varied each week.

Note that we incorporate vaccination into this model by removing, deterministically, a proportion of susceptibles to a separate vaccinated class (i.e., they are still counted as part of *N* but otherwise do not interact with the model dynamics). Specifically we remove 21% of the population, based on published vaccination rates and efficacy statistics in Australia [46, 47]. We chose to do this rather than take vaccination rate as a fitted parameter so as to minimize the number of parameters to be fit. Consequently, we set the prior for initial season susceptibility to be uniformly distributed from 0 to 0.75. We used temperature as the covariate with which to enable seasonal forcing, following the relationship $R_{0}={\bar{R}}_{0}+a\left(T-\bar{T}\right)$ (with *T* the daily mean temperature and $\bar{T}$ the mean temperature over the study period). A number of studies have considered a variety of climate covariates and their interaction with influenza transmission both in populations and experimental models (e.g., [48, 49]); with indications that relationships with and between climate factors are complex and may vary globally [50]. We noted similar quality model fits when using the specific humidity formulation presented by Shaman *et al*. [51], however we chose to use the simplest seasonal link, temperature, as this form produced similar quality fits while requiring fewer parameters and enabling greater ease of interpretation.

### Model fitting

We used approximate Bayesian computation [52, 53] to calculate posterior distributions for the model parameters. Specifically, we generated candidate parameter sets from the prior distributions listed above. For a given candidate parameter set, we simulated a realisation of the model, and observed the sample path (i.e., the number of infected individuals attending ASPREN doctors each week in the simulated realisation) for 2011 and 2013 (H1 seasons), or 2014 and 2016 (H3 seasons). We compared this simulated realisation to the true sample path, using the square root of mean squared error as score function

$$\begin{array}{c} D:=\sqrt{\frac{1}{\#\text{weeks}}\sum_{i=1}^{\#\text{weeks}}{\left({\text{true}}_{i}-{\text{candidate}}_{i}\right)}^{2}}, \end{array}$$

with true_*i*_ the observed number of cases in the *i*^*th*^ week from the ASPREN dataset and candidate_*i*_ the number in the simulated realisation from the candidate parameter set. There were 104 weeks in each of the study periods (# weeks). The parameter set is accepted if D is less than some tolerance level, in this case set to 4.5. We observe that this choice of score function and threshold produces reasonable model fits, while still being high enough to accept parameter sets regularly. Choice of score function in ABC is problem-specific [54], and it is likely that a variety of other metrics could reasonably be used to produce reasonable fits. Note that a candidate parameter set can produce a range of scores given that realisations are stochastic, and the accepted realisations are generally among the best possible realisations from the given parameter set (i.e., those that most closely fit the data), however, the average realisation from these parameter sets still fit the data relatively well (S1 Text).

Kernel density estimates were constructed from posterior samples using the algorithm of Botev *et al*. [55].

## Supporting information

S1 Text Supplemental information. Text includes a derivation for the transition rates, a full set of posterior parameter estimates, and details of some simulated examples.
(PDF)
