# Supplementary material for: Characterising seasonal influenza epidemiology using primary care surveillance data
Source: PLoS Comput Biol. 2018 Aug 16;14(8):e1006377. doi: 10.1371/journal.pcbi.1006377 (PMC6112683; doi:10.1371/journal.pcbi.1006377)
Supplement: S1 Text — Text includes a derivation for the transition rates, a full set of posterior parameter estimates, and details of some simulated examples. (PDF) [file pcbi.1006377.s001.pdf]

Characterising seasonal influenza epidemiology using primary care surveillance data – Supplementary Material

Robert C. Cope, Joshua V. Ross, Monique Chilver, Nigel P. Stocks, Lewis Mitchell

1 Parameter derivations

Model transitions out of the I_1 compartment depend upon two rates: 2γ and λ. An individual remains in this compartment for a time that is distributed $\text{Exp}(2\gamma + \lambda)$, i.e., the minimum of an $\text{Exp}(2\gamma)$ random variable and an $\text{Exp}(\lambda)$ random variable. The same holds for transitions out of compartment I_2. So, an individual that was not observed, spends on average $2/(2\gamma + \lambda)$ time units infectious.

We assume that an observed individual should on average spend the same amount of time infectious as an individual who is not observed. To this end, the rates out of O_1 and O_2 must be higher than 2γ, as these individuals undergo an additional $\text{Exp}(2\gamma + \lambda)$ time while transitioning from I to O. We denoted this rate 2α. We calculated the mean time an observed individual would spend infectious, given that they were observed on the first or the second transition (i.e., progressing through states $I_1 \to O_1 \to O_2 \to R$, or $I_1 \to I_2 \to O_2 \to R$, respectively). Using the probability of each of these transitions, we then calculate the overall mean time an observed individual would spend infectious. We equate the mean time that observed and unobserved individuals are infectious, allowing us to solve for α in terms of γ and λ. This results in $\alpha = 3\gamma + \lambda$.

We used the fitted parameters p_{obs} (the probability of observation), and D_{inf} (the mean time infectious) to derive the model parameters γ and λ. This involves solving two simultaneous equations:

$$p_{\text{obs}} = 1 - \left(\frac{2\gamma}{2\gamma + \lambda}\right)^2, \qquad D_{\text{inf}} = \frac{2}{2\gamma + \lambda},$$

resulting in:

$$\gamma = \frac{\sqrt{1 - p_{\text{obs}}}}{D_{\text{inf}}}, \qquad \lambda = \frac{2 - 2\sqrt{1 - p_{\text{obs}}}}{D_{\text{inf}}}.$$

2 Full model posterior distributions/outputs

In this section we present:

- The H3N2 strain equivalents to Figures 4 and 6 from the main text (Figure A, B).
- An example of how well model realisations generated from the prior fit the data (Figure C).
- A full set of marginal posterior density plots for H1N1pdm09 seasons (Figures D, E).
- A full set of marginal posterior density plots for H3N2 seasons (Figures F, G).
- The proportion of susceptible individuals that were infected in a season of a simulated realisation from each accepted parameter set (Figure H).

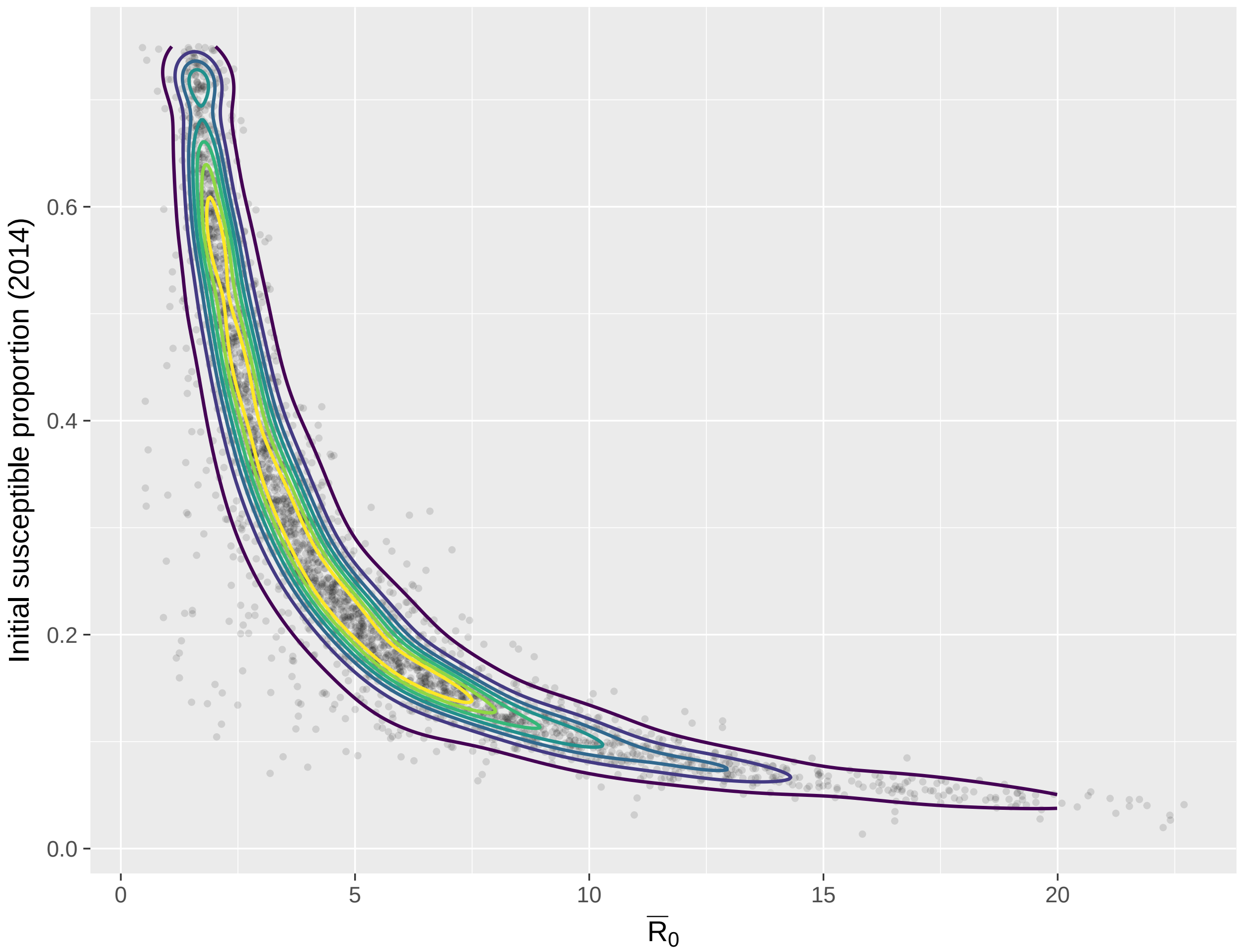

Figure A: Bivariate posterior distribution of $\bar{R}_0$ and initial susceptible proportion in 2014 for H3N2. Points indicate accepted ABC parameter sets. Contours indicate posterior credible intervals, such that each contour contains deciles of kernel-smoothed posterior probability density. The maximum bivariate posterior density occurred at $\bar{R}_0 = 4.71$ and initial susceptibility 0.22.

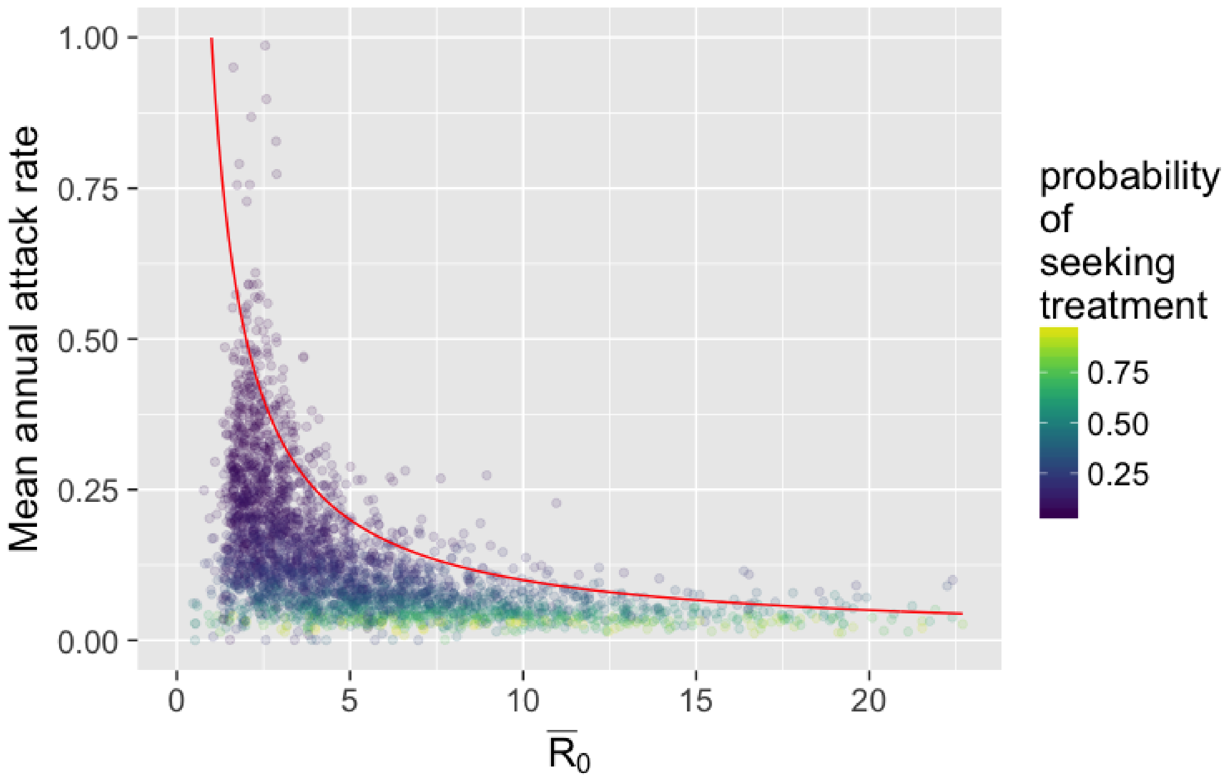

Figure B: The relationship between $\bar{R}_0$ and the population level attack rate of simulated realisations of the process, across 2014 and 2016 H3N2. For each accepted particle, the process was simulated again, and the total number of infected individuals from these new realisations was recorded, and converted into an annual attack rate (by dividing by 2 and the total population). Point colour indicates the probability of an individual seeking treatment; larger attack rates correspond to smaller treatment probabilities given that they all fit the same data. The red line shows denotes where the product of $\bar{R}_0$ and the attack rate would be equal to one; this is the line around which the initial population susceptibility values were situated. This can be interpreted as indicating that, for points under this line, not all susceptible individuals became infected during the season.

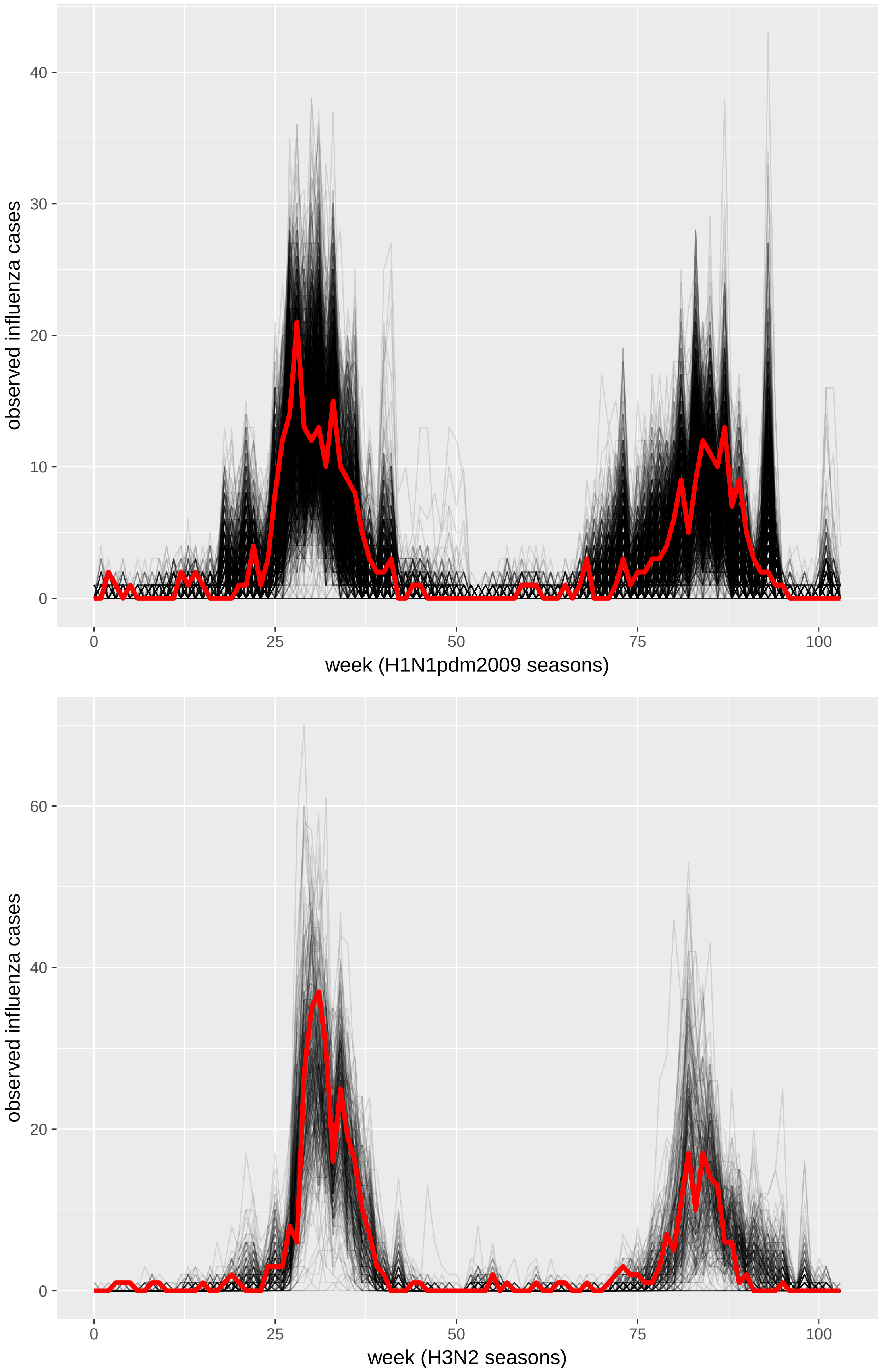

Figure C: Model realisations simulated from the posterior distribution (black lines), against the observed influenza data (red).

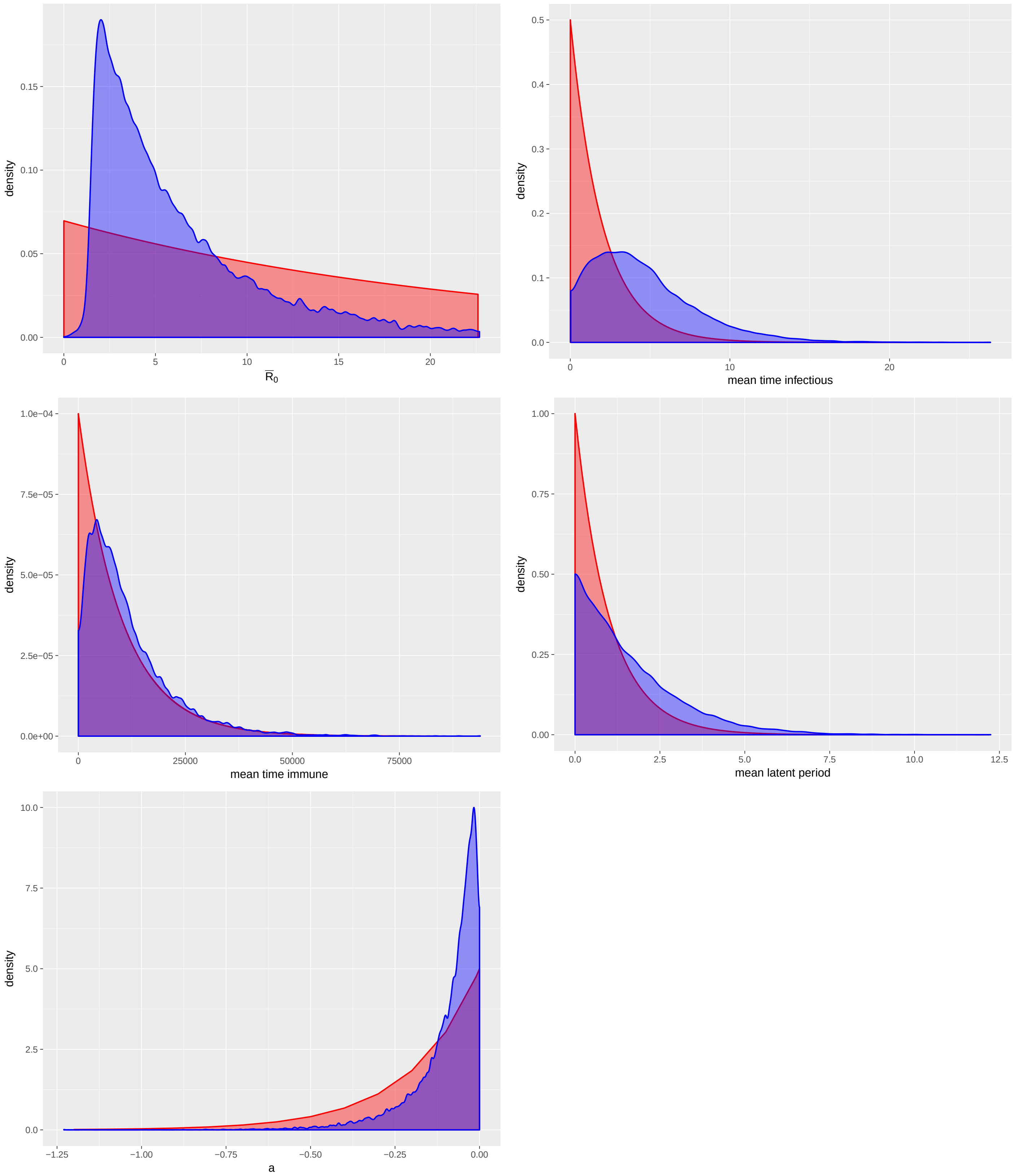

Figure D: Marginal posterior densities (blue) and prior distributions (red) for fitted parameters - H1N1pdm09 (2011 & 2013).

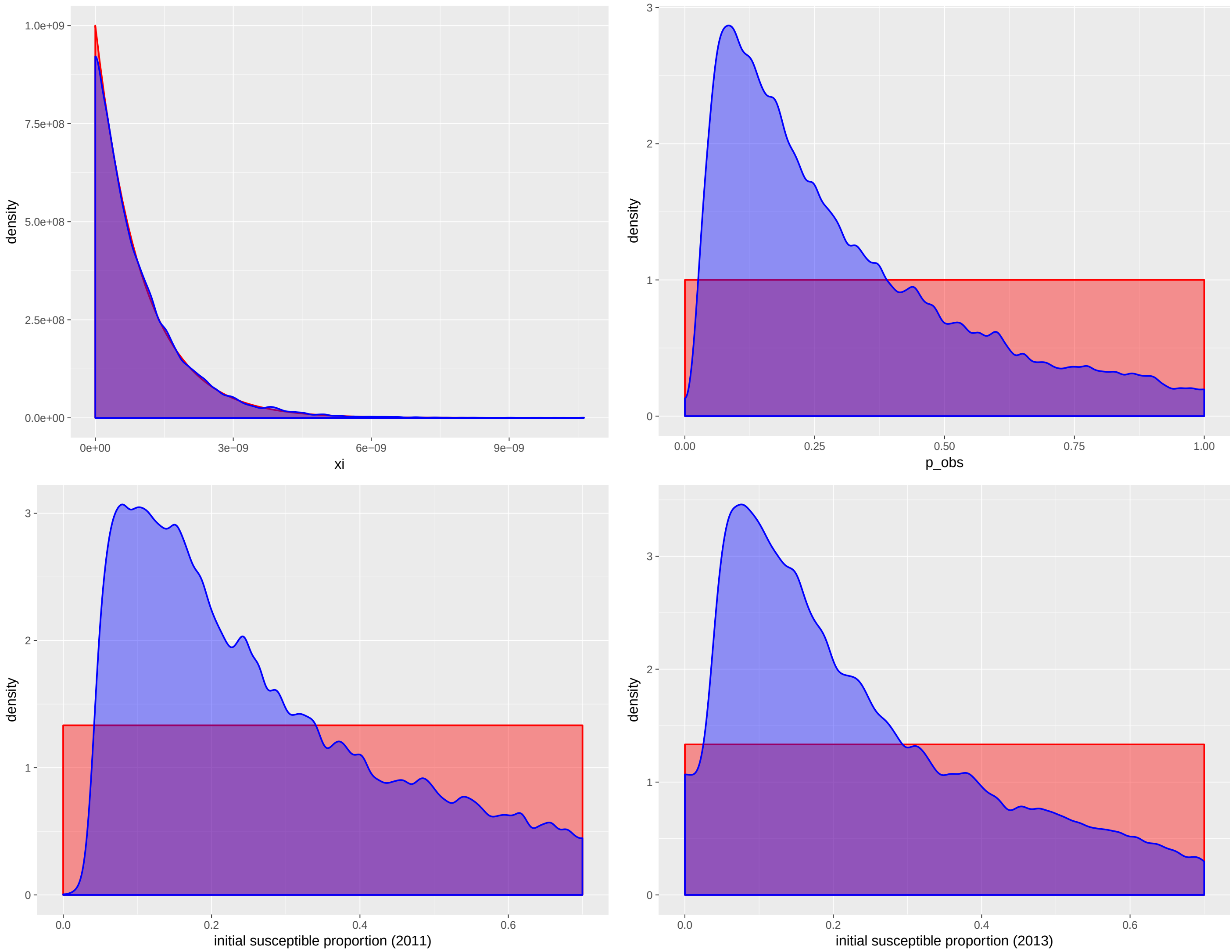

Figure E: Marginal posterior densities (blue) and prior distributions (red) for fitted parameters - H1N1pdm09 (2011 & 2013).

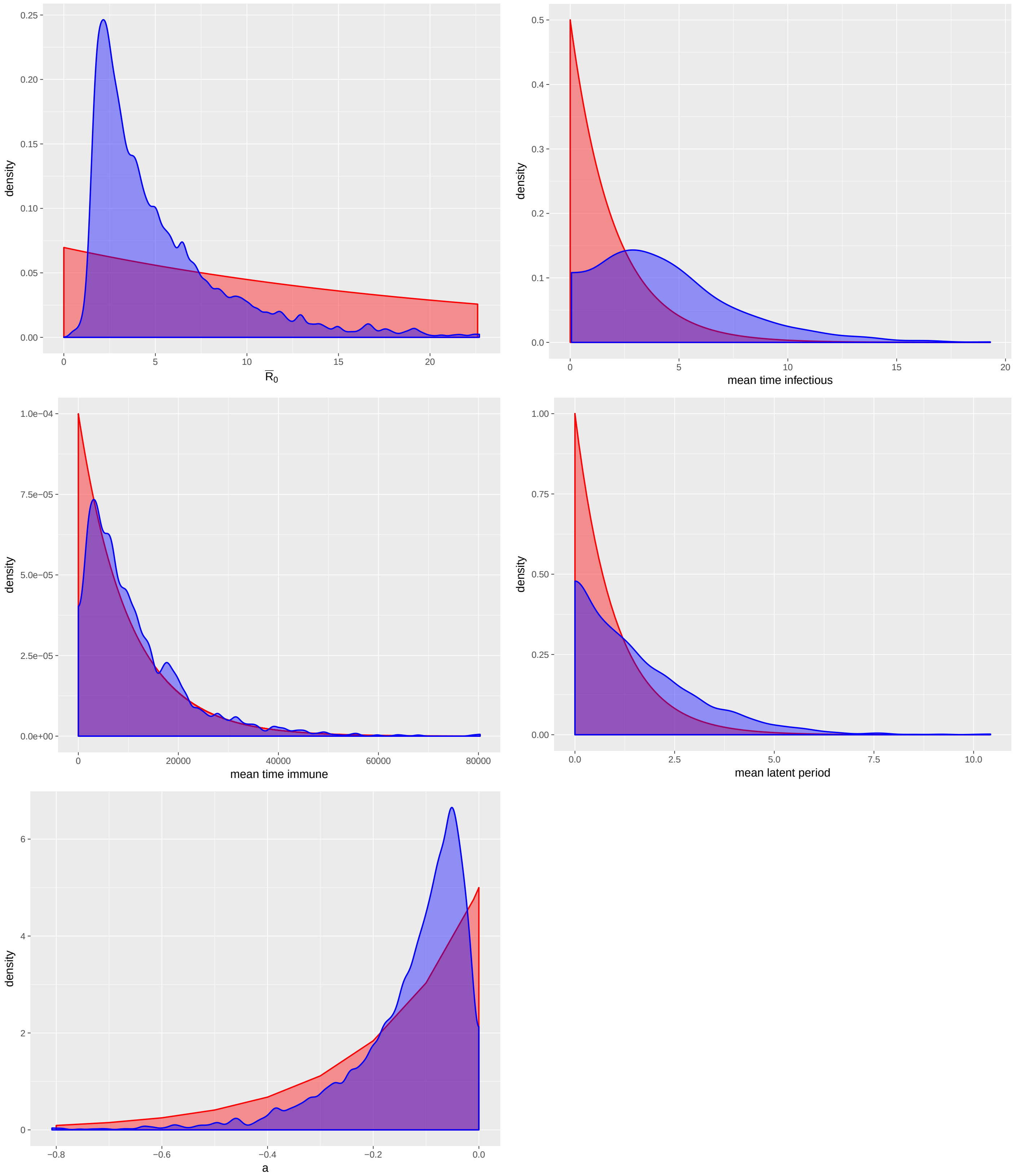

Figure F: Marginal posterior densities (blue) and prior distributions (red) for fitted parameters - H3N2 (2014 & 2016).

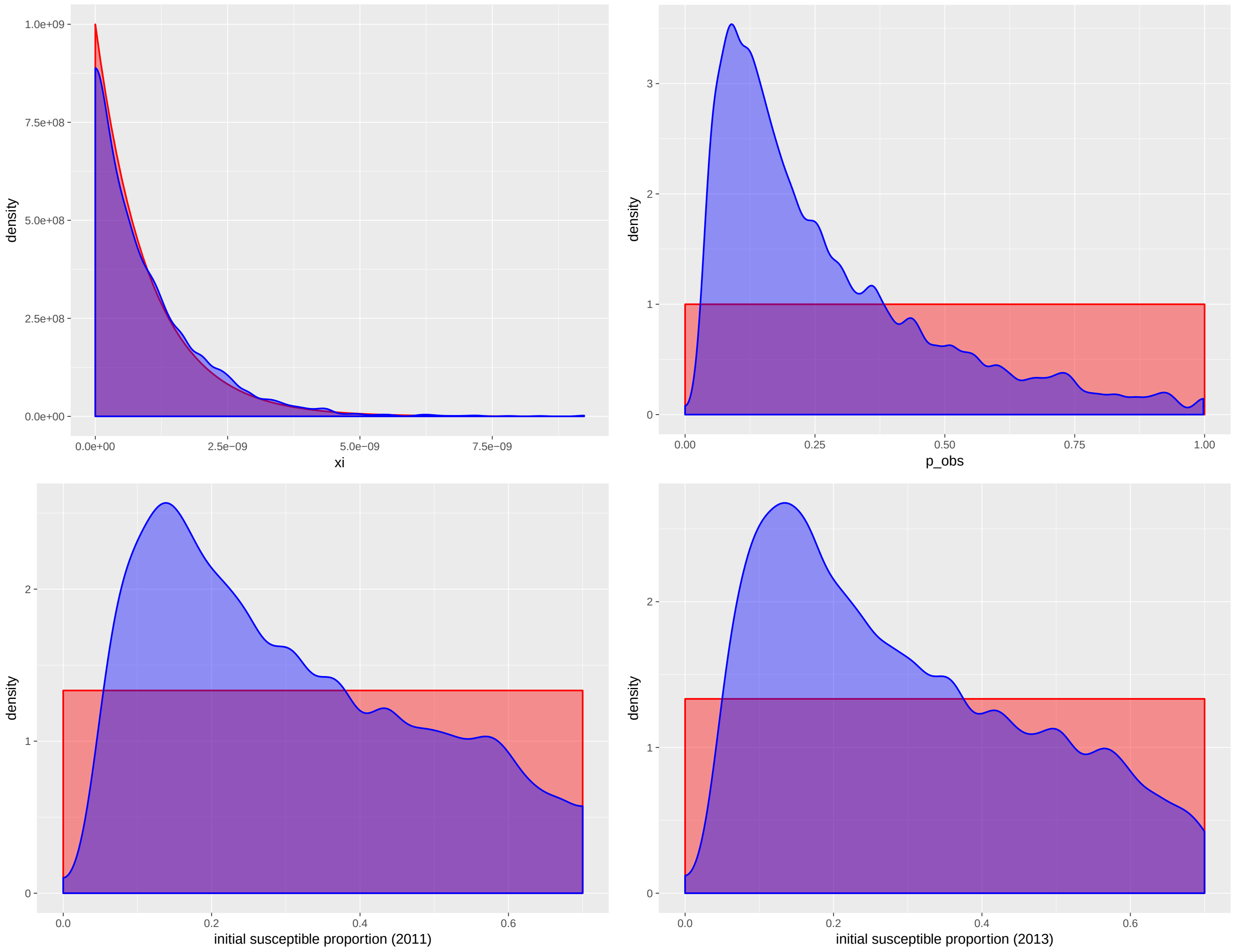

Figure G: Marginal posterior densities (blue) and prior distributions (red) for fitted parameters - H3N2 (2014 & 2016).

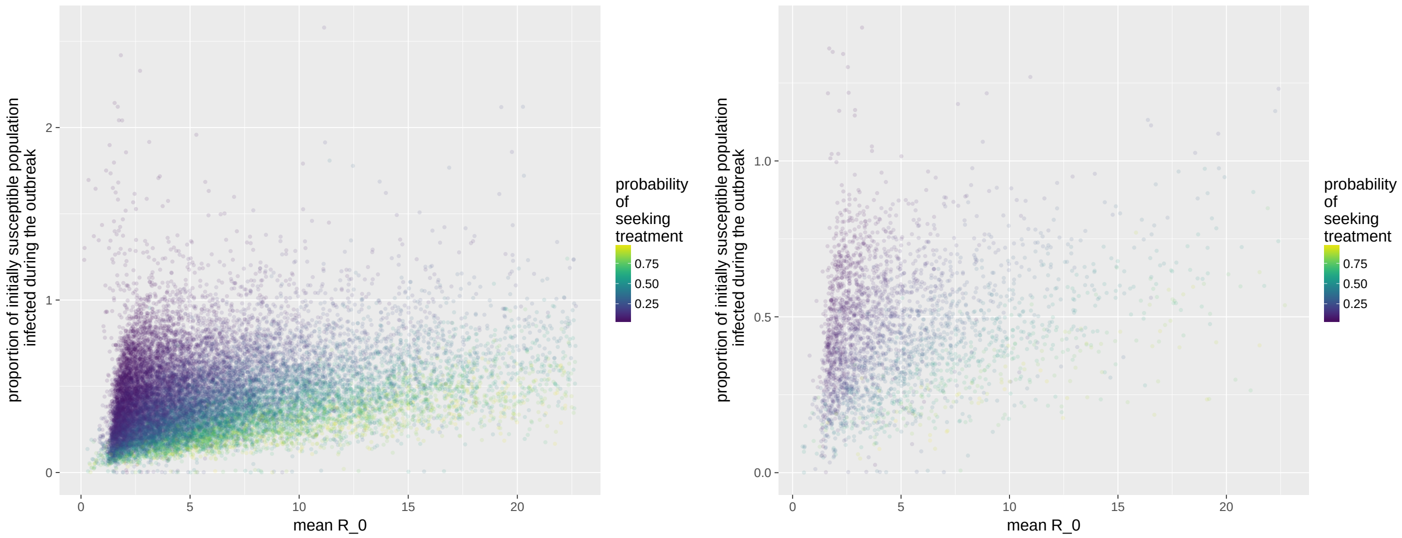

Figure H: Proportion of individuals susceptible in week 25 that were infected during a simulated realisation from each accepted posterior parameter set: (left) H1N1pdm09 (2011 & 2013), (right) H3N2 (2014 & 2016). The small number of parameter sets that exceed 1.0 indicate parameter sets with substantial amounts of with-season waning of immunity. For H1N1pdm09, the median proportion of susceptible infected during a season was 0.401 (90% CI 0.144–0.738). For H3N2, the median proportion of susceptible infected during a season was 0.439 (90% CI 0.164–0.703).

3 Simulated examples

To provide an illustrative example of the challenges of identifying parameters $\bar{R}_0$ and initial population susceptibility given the data observed, we simulated H1N1pdm09 epidemics (over both 2011 and 2013, and using the true climate observations and weekly sampling denominators from those years) from the model with parameters based upon accepted particles in different parts of the posterior distribution (Figure I). Note that the simulations shown are not the realisations that resulted in the particle being accepted, rather an additional realisation independent of the ASPREN data. The 100 particles closest to $\bar{R}_0 = 2,\ 6,\ 10,$ (with associated initial susceptibility of 0.6, 0.18, and 0.1, respectively) were chosen.

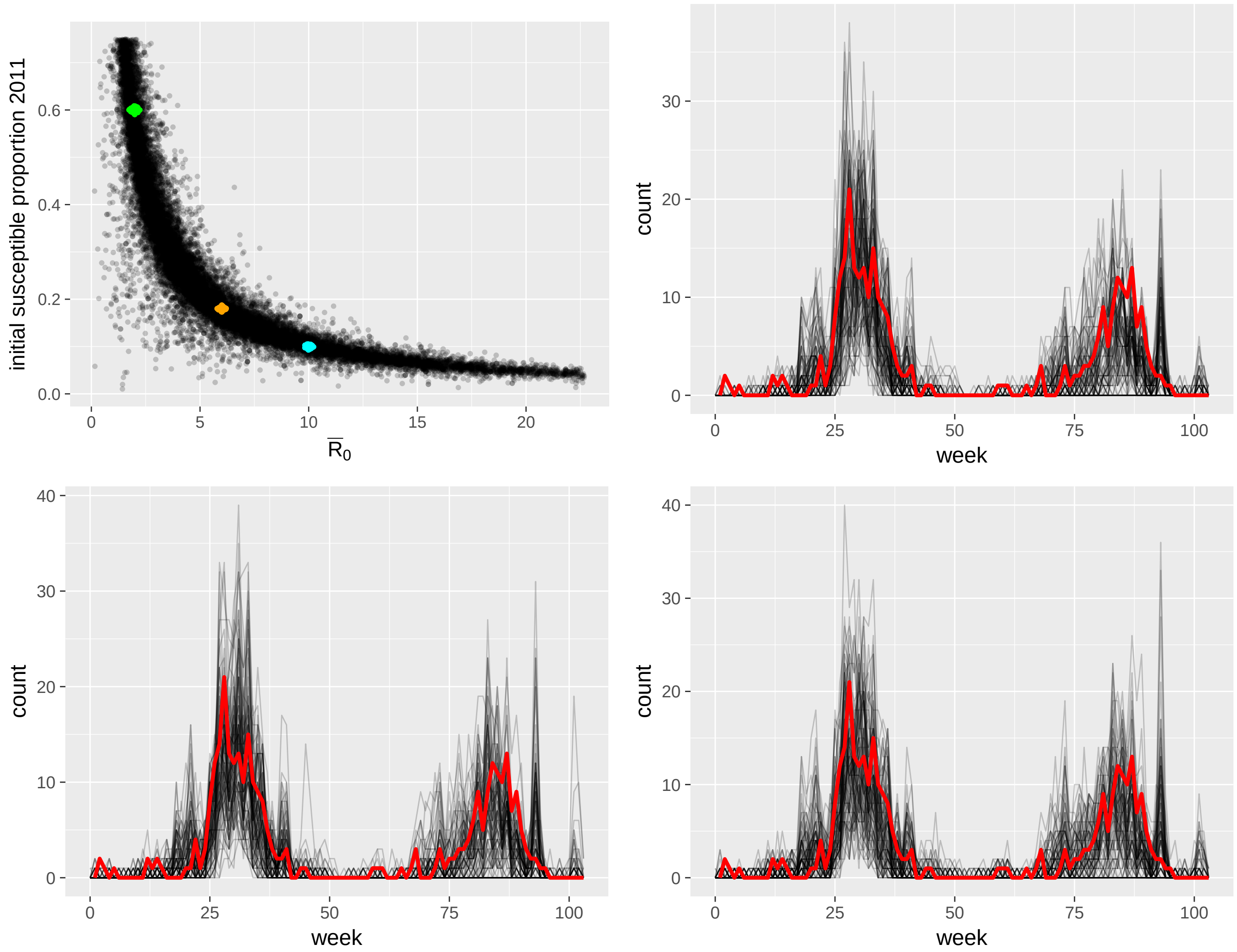

Figure I: Illustration of model simulations from different regions of the posterior distribution, demonstrating the challenges of identifying $\bar{R}_0$ and initial population susceptibility. (top left) The bivariate posterior distribution, with the location of three groups of posterior particles highlighted. (top right) Simulated realisations from each of 100 posterior particles around $\bar{R}_0 = 2$ and initial susceptibility 0.6. (bottom left) simulated realisations from each of 100 posterior particles around $\bar{R}_0 = 6$ and initial susceptibility 0.18. (bottom right) simulated realisations from each of 100 posterior particles around $\bar{R}_0 = 10$ and initial susceptibility 0.1. In each case, the red line shows the actual observed ASPREN data.
